# Application of amino acid occurrence for discriminating different folding types of globular proteins

**DOI:** 10.1186/1471-2105-8-404

**Published:** 2007-10-22

**Authors:** Y-h Taguchi, M Michael Gromiha

**Affiliations:** 1 Department of Physics, Faculty of Science and Technology, Chuo University, 1-13-27 Kasuga, Bunkyo-ku, Tokyo 112-8551, Japan; 2 Institute for Science and Technology, Chuo University, 1-13-27 Kasuga, Bunkyo-ku, Tokyo 112-8551, Japan; 3 Computational Biology Research Center (CBRC), National Institute of Advanced Industrial Science and Technology (AIST), AIST Tokyo Waterfront Bio-IT Research Building, 2-42 Aomi, Koto-ku, Tokyo 135-0064, Japan

## Abstract

**Background:**

Predicting the three-dimensional structure of a protein from its amino acid sequence is a long-standing goal in computational/molecular biology. The discrimination of different structural classes and folding types are intermediate steps in protein structure prediction.

**Results:**

In this work, we have proposed a method based on linear discriminant analysis (LDA) for discriminating 30 different folding types of globular proteins using amino acid occurrence. Our method was tested with a non-redundant set of 1612 proteins and it discriminated them with the accuracy of 38%, which is comparable to or better than other methods in the literature. A web server has been developed for discriminating the folding type of a query protein from its amino acid sequence and it is available at http://granular.com/PROLDA/.

**Conclusion:**

Amino acid occurrence has been successfully used to discriminate different folding types of globular proteins. The discrimination accuracy obtained with amino acid occurrence is better than that obtained with amino acid composition and/or amino acid properties. In addition, the method is very fast to obtain the results.

## Background

Deciphering the native conformation of a protein from its amino acid sequence, known as, protein folding problem is a challenging task. The recognition of proteins belonging to same fold/structural class is an intermediate step to protein structure prediction. For the past few decades, several methods have been proposed for predicting protein structural classes. These methods include discriminant analysis [1], correlation coefficient [2], hydrophobicity profiles [3], amino acid index [4], Bayes decision rule [5], amino acid distributions [6], functional domain occurrences [7], supervised fuzzy clustering approach [8] and amino acid principal component analysis [9]. These methods discriminated protein structural classes with the sensitivity of 70–100% and it mainly depends on the data set. Wang and Yuan [5] developed a data set of 674 globular protein domains belonging to four different structural classes and reported that methods claiming 100% sensitivity for structural class prediction could predict only with the sensitivity of 60% with this data set.

On the other hand, alignment profiles have been widely used for recognizing protein folds [10,11]. Recently, Cheng and Baldi [12] proposed a machine learning algorithm for fold recognition using secondary structure, solvent accessibility, contact map and *β*-strand pairing, which showed a pair wise sensitivity of 27%. On the other hand, amino acid properties have been used for discriminating membrane proteins [13], identification of membrane spanning regions [14], prediction of protein structural classes [15], protein folding rates [16], protein stability [17] etc. Towards this direction, Ding and Dubchak [18] proposed a method based on neural networks and support vector machines for fold recognition using amino acid composition and five other properties, and reported a cross-validated sensitivity of 45%. Further, Ofran and Margalit [19] showed the existence of significant similarity in amino acid composition between proteins of the same fold. In this work, we have used amino acid occurrence (not composition) for discriminating 30 different folding types of globular proteins. We have developed a method based on linear discriminant analysis (LDA), which discriminated a set of 1612 proteins with an accuracy of 38%, which is comparable to other methods in the literature, in spite of the simplicity of the method and large dataset.

## Results and discussion

### Role of re-weighting for fold discrimination

We have computed the occurrence of all the 20 amino acid residues in each protein, which represents the elements of 20 dimensional vectors in each protein. We have applied LDA to these vectors for discrimination. We have employed two kinds of LDA, i.e., with and without re-weighting. In LDA with re-weighting, i.e. *W*_*k *_= 1 in eq. (1), all folds equally contribute to maximize the performance of discrimination irrespective of the number of proteins in each fold; i.e., if one fold has hundreds of proteins and another has only few proteins, LDA is optimized to achieve the highest performance equally in all folds. This re-weighting is important especially when the number of proteins included in each fold has large variations. On the other hand, LDA without re-weighting, i.e. *W*_*k *_= *N*_*k *_in eq. (1), tends to achieve the maximum performance for the whole dataset.

We have used the measures, accuracy, sensitivity, precision and F1 for examining the performance of the method. In general, accuracy has the tendency to show high values without re-weighting since it is computed with all data. Sensitivity tends to increase by re-weighting, giving equal weight to each fold. In contrast, precision has the tendency to decrease by re-weighting, since re-weighting increase FP for folds with less number of proteins. On the other hand, F1 is independent of re-weighting as it is harmonic mean of sensitivity and precision.

In Table 1, we presented the discrimination results obtained with different measures and two kinds of LDA (with and without re-weighting). As expected, re-weighting significantly changed all the performances other than F1. Re-weighting increased the sensitivity whereas an opposite trend was observed for precision and accuracy. This is due to the divergence in the number of proteins in each fold (min. 25, max. 173, mean 54, see Table 2). F1 does not change significantly by re-weighting.

**Table 1:** Role of re-weighting. Leave-one-out cross validation results [%] obtained with different measures and two types of LDA

	with re-weighting				without re-weighting			
	
	sensitivity	precision	F1	accuracy	sensitivity	precision	F1	accuracy
Occurrence	33	29.	29	33	28	35	30	38
Composition	27	23	23	26	24	27	27	33

**Table 2:** Performances of fold recognition. Leave-one-out cross validation performances [%] in each fold. wo: without re-weighting, w: with re-weighting

						Sensitivity		Precision		F1	
						
ID	Fold	Fold Description		Number	Ratio	wo	w	wo	w	wo	w
		all-*α*									
1	a.3	Cytochrome C		25	2	24	48	50	27	32	35
2	a.4	DNA/RNA binding 3-helical bundle		103	6	73	49	43	51	54	50
3	a.24	Four helical up and down bundle		26	2	23	38	35	20	28	26
4	a.39	EF hand-like fold		25	2	40	44	45	26	43	33
5	a.60	SAMdomain-like		26	2	8	27	29	12	12	16
6	a.118	*α*-*α *superhelix		47	3	47	45	50	50	48	47
		all-*β*									
7	b.1	Immunoglobulin-like *β*-sandwich		173	11	76	38	41	69	54	49
8	b.2	Common fold of diphtheria toxin/transcription factors/cytochrome f		28	2	4	29	11	21	5	24
9	b.6	Cupredoxin-like		30	2	27	37	42	22	33	27
10	b.18	Galactose-binding domain-like		25	2	20	36	50	26	29	30
11	b.29	Concanavalin A-like lectins/glucanases		26	2	23	27	24	18	24	22
12	b.34	SH3-like barrel		42	3	0	29	0	20	-	24
13	b.40	OB-fold		78	5	22	24	24	24	23	24
14	b.82	Double-stranded *α*-helix		34	2	12	18	19	17	15	17
15	b.121	Nucleoplasmin-like		42	3	52	52	51	47	52	49
		*α*/*β*									
16	c.1	TIM barrel		145	9	44	27	57	65	50	38
17	c.2	NAD(P)-binding Rossmann-fold domains		77	5	34	31	30	32	32	32
18	c.3	FAD/NAD(P)-binding domain		31	2	10	16	13	11	11	13
19	c.23	Flavodoxin-like		55	3	11	5	17	8	13	7
20	c.26	Adenine nucleotide a hydrolase-like		34	2	12	29	14	22	13	25
21	c.37	P-loop containing nucleoside triphosphate hydrolases		95	6	43	34	42	53	43	41
22	c.47	Thioredoxin fold		32	2	9	19	38	10	15	13
23	c.55	Ribonuclease H-like motif		49	3	4	6	11	8	6	7
24	c.66	S-adenosyl-L-methionine-dependent methyltransferases		34	2	29	29	31	21	30	24
25	c.69	*α*/*β*-Hydrolases		37	2	35	41	39	34	37	37
		*α *+ *β*									
26	d.15	*β*-Grasp, ubiquitin-like		42	3	5	21	40	18	9	19
27	d.17	Cystatin-like		25	2	0	8	-	4	-	5
28	d.58	Ferredoxin-like		118	7	32	7	17	25	22	11
		small									
29	g.3	Knottins		80	5	98	89	72	82	83	85
30	g.41	Rubredoxin-like		28	2	11	71	75	32	19	44

Remarkably, we achieved the accuracy of 38% (without re-weighting), which is the best performance to our knowledge, for large number of folds (30) and proteins (1612). Further, the method is extremely simple, which indicates that the amino acid occurrence of proteins carry sufficient information to discriminate protein folds.

### Discrimination of proteins belonging to different folding types

We have examined the ability of the present method for predicting proteins belonging to 30 major folds. In Table 2, we have shown the performances of discriminating 30 different folds. We observed that the folds with less number of proteins have the sensitivity of less than 10% without re-weighting. For example, SAM domain like fold has the sensitivity of 8%, which has only 26 proteins. Similar tendency is also observed for the folds b.2, b.34, c.3, c.47, c.55, d.15 and d.17. The sensitivity of these folds increased significantly with re-weighting. On the other hand, many folds with less than 30 proteins have the sensitivity of more than 20% without re-weighting (e.g., a.3, a.24, a.39 etc.). As there are 30 folds, the expected sensitivity is only 3.3% if classification is supposed to be random. In Table 2, we have also shown the ratio between the number of proteins in each fold and the total number of proteins, which ranges from 2 to 11%. Hence the sensitivity of 20% obtained for several folds is significantly higher than that of random for fold discrimination. Interestingly, most of the folds that were discriminated with more than 20% sensitivity belong to either all-*α *or all-*β *class. This might be due to the fact that these proteins have different secondary structural patterns and hence they are easy to discriminate them. In addition, folds in each of these classes are near-by each other in amino acid occurrence vector space, which caused high sensitivity. On the other hand, an opposite tendency was observed for precision. Re-weighting decreased the precision for several folds including a.3, a.24, a.29, a.60, b.6, b.18, b.29, c.23, c.47, d.15, and g.41. Most of these folds have less number of proteins.

The re-weighting procedure causes two opposite effects: increased the sensitivity and decreased the precision. Hence, F1 may be used to balance these effects. Only two folds, c.23 and d.58 decreased the F1 with re-weighting and several folds significantly increased the F1 by re-weighting (e.g, b.2, b.34, c.26, d.17, and g.41).

**Figure 1:**
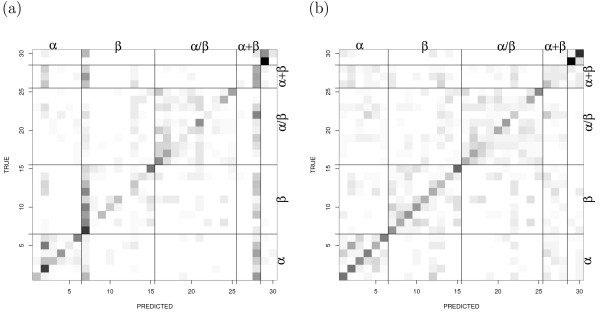
**Prediction versus experiment**. Comparison between predicted and experimental folds in 1612 proteins. The diagonal elements show the correctly predicted proteins. Dark block indicates the presence of more number of proteins and solid line indicates the boundary between five classes as shown in Table 2, i.e., all-*α*, all-*β*, *α*/*β*, and *α *+ *β *and small proteins. (a)without re-weighing. (b) with re-weighing.

The comparison between experimental versus predicted folds is shown in Fig. 1. In this figure, dark block indicates the presence of many proteins. The data are normalized in such a way that the total percentage of true fold is 100%. Fig. 1a showed that mainly the folds with more number of proteins are misclassified without re-weighting (e.g., a.4, b.1, c.1 and d.58). The trend has been changed after re-weighting: the misclassified proteins are observed to be within the same structural class. Especially, in *α *+ *β *class, the block diagonal region is distributed almost uniformly, which is partially caused by re-weighting. Since each fold is equally weighted, *α *+ *β *class is less weighted than other classes. This causes inter-class misclassification between *α *+ *β *and other classes, because *α *+ *β *class has only three folds. However, two folds in small structural class can be discriminated with high accuracy/sensitivity/precision/F1 and *α *+ *β *folds are difficult to discriminate using our method.

### Comparison among different re-weighting procedures

The results presented in Tables 1 and 2 showed that the sensitivity of discriminating protein folds differs significantly between different methods (with and without re-weighting). Hence, it would be difficult to choose the best method for fold recognition. However, it may be selected based on the interest of the users, whether the prediction is optimized for each fold or over all dataset.

Usually, training and test sets of data are obtained from sequence and structure databases and are culled with sequence identity. However, these data sets do not always reflect proper representatives of all proteins in different folds, e.g., protein population in each fold. Further, the proteins available in databases such as, PDB are biased with the proteins that can be solved experimentally, which may be different from the proportion of real proteins. Hence, considering these aspects would help to develop "good" methods for protein fold recognition in future.

In essence, based on the methods and data sets used in the present work, we suggest that the performance with re-weighting is better than that without re-weighting.

### Influence of amino acid occurrence in recognizing protein folds

**Figure 2:**
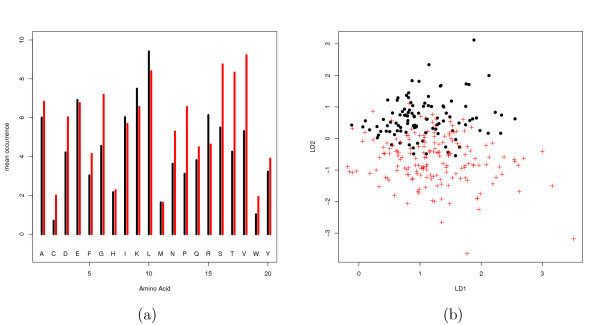
**Amino acid occurrence**. (a)Comparison between mean amino acid occurrence of two typical folds, DNA/RNA binding *α*-helical bundle (a.4, black) and Immunoglobulin-like *β*-sandwich (b.2, red) (b) Distribution of these two folds over the first two discriminant functions with re-weighting. a.4: filled black circles, b.2: red crosses.

The importance of amino acid occurrence is illustrated with Figure 2(a). In this figure we show the occurrence of the 20 types of amino acid residues in DNA/RNA binding 3-helical bundle (a.4) and Immunoglobulin-like *β*-sandwich (b.1). The average number of amino acid residues in these folds are 88 and 110, respectively. We observed that the residues Gly, Pro, Ser, Thr and Val are dominant in the fold b.1 whereas an opposite trend was observed for Leu and Arg. In Figure 2(b), we have shown the distribution of residues in "amino acid occurrence" space. It is clearly seen that the two folds are more or less separated in this space. We observed similar results about the variation of amino acid occurrences among different folds in our data set.

In addition, we have tested the performance of the method using amino acid composition (i.e., amino acid occurrence/total number of residues) in each protein. We noticed that the accuracy without re-weighting decreased to 33% indicating the importance of amino acid occurrence (un-normalized composition) in each fold (Table 1). Similar tendency is also observed for discriminating *β*-barrel membrane proteins [16]. Hence, we suggest that the amino acid occurrence is better than composition for discriminating protein folds. In fact, the normalization of amino acid composition produced the problem of co-linearity, i.e., diversity of vectors is not sufficient compared with the number of proteins. The reason for the dependency of F1 upon different types of LDA (with or without re-weighting) is that four folds have no positive proteins without re-weighting. On the other hand, amino acid occurrence has only two folds without any positive proteins (without re-weighting) as seen in Table 2.

### Probability measure of discrimination

**Figure 3:**
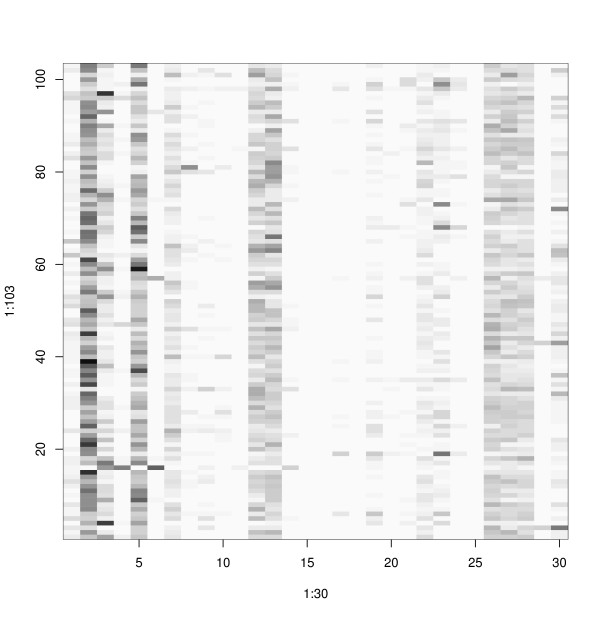
**Probability measure of discrimination**. Rows : 103 proteins in fold (a.4). Columns : 30 folds. From left to right, the order is ID in Table 2. The darkest square corresponds to probability 0.5, and the lightest is zero.

In order to have the feasibility of combining the results of our method with other methods we provided the probability of being a protein in a specific fold along with the predicted folding type. In Figure 3, we have shown the probability for fold a.4 (DNA/RNA binding 3-helical bundle). Clearly, the fold a.4 has the highest average probability. However, some other folds, (e.g., a.60, d.15, d.17 and d.58) have relatively higher probabilities. This may result in wrong discrimination, which may be fixed by combining the results with other methods.

### Comparison with other methods

We have compared the performance of our method with other related works in the literature. Ding and Dubchak [18] introduced a combined method for predicting the folding type of a protein. They have used six parameters, amino acid composition, secondary structure, hydrophobicity, van der Waals volume, polarity and polarizability as attributes, and neural networks and support vector machines for recognition. These features have been combined with the number of votes in each method. They reported the sensitivity of 56% in a test set of 384 proteins and 10-fold cross validation sensitivity of 45% in a training set of 311 proteins from 27 folding types. We have used the same dataset of 311 proteins and assessed the performance of our method. We observed that our method could predict with the leave-one-out cross validation accuracy of 42% (with LDA without re-weighting), which is close to that (45%) reported in Ding and Dubchak [18].

In addition, we have selected the proteins from the folds that are common in both the studies and tested the performance of our method (trained with our dataset of 1612 proteins) in predicting the folding types of the proteins used in Ding and Dubchak [18]. The results are presented in Table 3. Interestingly, our method with re-weighting could correctly identify the folding types with F1 value of more than 30% in 11 among the 19 considered folds. The performances are similar to or better than that reported with the dataset of 1612 proteins. Although our method is optimized with different datasets it has the power to predict the folding type of independent dataset of proteins with similar sensitivity.

**Table 3:** Performances with independent dataset Predictive ability [%]of our method to the independent dataset of proteins used in Ding and Dubchak [18]. wo: without re-weighting, w: with re-weighting

	Number	Ratio	Sensitivity		Precision		F1	
			
Fold Description		[%]	wo	w	wo	w	wo	w
Cytochrome C	16	3	56	94	64	47	60	63
DNA/RNA binding 3-helical bundle	32	6	75	56	41	47	53	51
Four helical up and down bundle	15	3	33	33	71	42	45	37
EF hand-like fold	15	3	53	53	57	42	55	47
Immunoglobulin-like *β*-sandwich	74	14	66	31	44	68	53	43
Cupredoxin-like	21	4	29	38	50	33	36	36
Concanavalin A-like lectins/glucanases	13	2	38	38	42	33	40	36
SH3-like barrel	16	3	0	50	-	44	-	47
OB-fold	32	6	16	28	26	31	20	30
TIM barrel	77	14	40	25	66	70	50	37
FAD/NAD: (P)-binding domain	23	4	22	30	114	50	37	38
Flavodoxin-like	24	5	8	13	28	35	13	18
NAD: (P)-binding Rossmann-fold domains	40	8	40	35	5	8	8	13
P-loop containing nucleoside triphosphate hydrolases	22	4	23	18	38	50	29	27
Thioredoxin fold	17	3	18	35	33	25	23	29
Ribonuclease H-like motif	22	4	5	18	14	22	7	20
*α*/*β*-Hydrolases	18	3	33	39	43	41	38	40
*β*-Grasp, ubiquitin-like	15	3	0	33	0	20	-	25
Ferredoxin-like	40	8	23	3	11	10	15	4

Total/Mean	532		31	35	42	38	34	34

Accuracy								
							
without reweighting	36							
with reweighting	32							

Further, there are several advantages in our method: (i) only one feature, amino acid occurrence is sufficient for prediction rather than six features. The comparison of results obtained with only one feature showed that the performance of our method (42%) is significantly better than that of Ding and Dubchak [18] reported with amino acid composition (20–49%), (ii) voting procedure is not necessary; our method can be directly used for multi-fold classifications, (iii) our method uses LDA, which requires significantly less computational power compared with SVM. In SVM one has to diagonalize the matrix with the size of (protein number) × (protein number); on the other hand, LDA requires only diagonalization of 20 (the number of kinds of amino acid residues) × 20 matrix independent of number of proteins and (iv) although they have reported the dependency of fold specific sensitivities upon number of proteins in each fold, it is difficult to compensate this effect without modifying the complicated voting systems; our method has freedom to compensate it as discussed in the previous sections.

Recently, Shen and Chou [20] reported better sensitivity for the same data set of Ding and Dubchak [18]. However, the results are biased with training set of data. We have evaluated the sensitivity of identifying proteins belonging to the folds, four helical up and down bundle (a.24) and EF hand-like (a.39) and we observed that the sensitivity is 30.5% and 24%, respectively. Our predicted sensitivities (38% and 44%, with re-weighting, see table 2) are better than that of Shen and Chou [20].

### Possible reasons for obtaining good performance with amino acid occurrence

We have analyzed the possible reasons for obtaining good performance with amino acid occurrence. In Table 4, we have summarized the performance as a function of different features. When we use more than two features to discriminate folds, we simply apply LDA to merge feature vectors. This means, if there are two features vectors ${\vec{f}}_{n}$ with *n *components and ${\vec{f}}_{m}$ with *m *features,

**Table 4:** Performances with other features Mean performances [%] obtained with different features for the data set used in Ding and Dubchak [18]. Re-weighting scheme is employed

	Sensitivity	Precision	F1	Accuracy
	Features			
	
secondary structure	35	32	40	36
polarity	19	18	26	21
polarizability	18	18	26	19
hydrophobicity	23	22	28	24
volume	21	20	25	22

	Composition			
	
composition	34	33	34	35
composition + length	36	35	38	38
composition + other five features	35	39	39	39

	Occurrence			
	
occurrence	40	40	39	42
occurrence + other five features	40	46	42	44

$${\vec{f}}_{m}=(f_{m1},f_{m2},...,f_{mm}),$$

then we merge and apply LDA to

$${\vec{f}}_{m+n}=(f_{n1},f_{n2},...,f_{nn},f_{m1},f_{m2},...,f_{mm}).$$

The usage of five features, i.e., predicted secondary structure, hydrophobicity, normalized van der Waals volume, polarity, polarizability [18] along with amino acid composition yielded the accuracy of 45% using sophisticated and time consuming methods. On the other hand, our simple method employing amino acid occurrence and five features has also showed almost the same value (44%).

The in-depth analysis of the results presented in Table 4 revealed many interesting features. For example, amino acid composition alone showed the accuracy of 35%, which is 7% less than that obtained with occurrence (42%). On the other hand, composition and length (i.e., the first 20 components of feature vectors consist of composition and the 21th component is amino acid length) increased the accuracy from 35% to 38%. The composition and five features showed the accuracy of 39%, which is similar (38%) to that obtained with composition and length. Hence, length of the protein has an important role as that of five features for discriminating protein folds. This analysis demonstrates the importance of amino acid length and obtaining good performance with amino acid occurrence.

As an individual feature amino acid occurrence showed the best performance among all features, including secondary structure. The combination of amino acid occurrence with other features did not increase the sensitivity and the increase of other parameters is only marginal. This result reveals that the amino acid occurrence contains most of the information that are reflected in other physical features.

Generally, *any *physical feature can be expressed by amino acid occurrence. Hence, linear combination of amino acid occurrence may express many of physical properties of proteins. In order to verify this concept, we have computed the correlation coefficients between 49 amino acid properties [21-23] and the first discriminate function. Each property consists of 20 dimensional vector, like

$${\tilde{P}}^{k}=(P_{1}^{k},P_{2}^{k},...,P_{i}^{k},...,P_{20}^{k}),$$

where $P_{i}^{k}$ is the *k*th physical property of *i*th amino acid. Since discriminant function is also 20 dimensional vector and each component of which describes contribution from each amino acid, one can compute correlation coefficient between them.

As can be seen in Table 5, 23 out of 49 properties have high correlation coefficients and less than 5% *q*-values (i.e., FDR corrected *p*-values). This analysis shows that linear discriminant function can express many of physical properties, at least, partly. Hence, even if we do not consider physical properties directly, the consideration of amino acid occurrence could discriminate folds well.

**Table 5:** Correlation between physical properties and the first discriminant function Brief descriptions of 49 selected physico-chemical, energetic and conformational properties, their correlation coefficient with the first discriminate function, and *q*-value. Asterisks in the last column shows *q*-value is less than 5%

No.	Description	Corr. Coef.	*q*-value [%]	*q *≤ 5%
1.	Compressibility	0.04	38.6	
2.	Thermodynamic transfer hydrophobicity	0.54	1.9	*
3.	Surrounding hydrophobicity	0.74	0.4	*
4.	Polarity	0.36	9.2	
5.	Isoelectric point	0.02	41.2	
6.	Equilibrium constant with reference to the ionization property	0.01	41.7	
7.	Molecular weight	0.06	38.4	
8.	Bulkiness	0.49	3.0	*
9.	Chromatographic index	0.51	2.7	*
10.	Refractive index	0.36	9.2	
11.	Normalized consensus hydrophobicity	0.48	3.4	*
12.	Short and medium range non-bonded energy	0.11	32.7	
13.	Long-range non-bonded energy	0.65	0.7	*
14.	Total non-bonded energy	0.57	1.5	*
15.	Alpha-helical tendency	0.29	14.1	
16.	Beta-helical tendency	0.63	0.8	*
17.	Turn tendency	0.61	0.9	*
18.	Coil tendency	0.60	1.1	*
19.	Helical contact area	0.20	23.0	
20.	Mean rms fluctuational displacement	0.57	1.5	*
21.	Buriedness	0.63	0.8	*
22.	Solvent accessible reduction ratio	0.70	0.4	*
23.	Average number of surrounding residues	0.72	0.4	*
24.	Power to be at the N-terminal of alpha helix	0.57	1.5	*
25.	Power to be at the C-terminal of alpha helix	0.18	26.4	
26.	Power to be at the middle of alpha helix	0.05	38.6	
27.	Partial-specific volume	0.25	18.8	
28.	Average medium-range contacts	0.11	32.7	
29.	Average long-range contacts	0.65	0.7	*
30.	Combined surrounding hydrophobicity (globular and membrane)	0.69	0.4	*
31.	Solvent accessible surface area for denatured protein	0.12	32.7	
32.	Solvent accessible surface area for native protein	0.52	2.5	*
33.	Solvent accessible surface area for protein unfolding	0.47	3.7	*
34.	Gibbs free energy change of hydration for unfolding	0.30	14.1	
35.	Gibbs free energy change of hydration for denatured protein	0.40	7.3	
36.	Gibbs free energy change of hydration for native protein	0.46	4.1	*
37.	Unfolding enthalpy change of hydration	0.05	38.6	
38.	Unfolding entropy change of hydration	0.37	8.9	
39.	Unfolding hydration heat capacity change	0.54	1.9	*
40.	Unfolding Gibbs free energy change of chain	0.16	27.6	
41.	Unfolding enthalpy change of chain	0.22	21.7	
42.	Unfolding entropy change of chain	0.44	4.7	*
43.	Unfolding Gibbs free energy change	0.33	11.0	
44.	Unfolding enthalpy change	0.35	10.2	
45.	Unfolding entropy change	0.34	10.3	
46.	Volume (number of non-hydrogen side chain atoms)	0.11	32.7	
47.	Shape (position of branch point in a side-chain)	0.10	32.8	
48.	Flexibility (number of side-chain dihedral angles)	0.24	19.5	
49.	Backbone dihedral probability	0.51	2.5	*

### Fold recognition on the web

We have developed a web server for discriminating protein folds from amino acid sequence [24]. It takes the amino acid sequence as input and displays the folding type in the output along with probability. Further, the server has the feasibility of selecting the method, with and without re-weighting, and the display options to show the probability details for each fold.

### Advantages and limitations of the method

The main advantage of the present method is the discrimination of 30 different folding types of globular proteins with high accuracy/sensitivity/precision/F1. Further it will provide the probability of being a protein in a specific fold. The discrimination results along with probability may be helpful to select templates to build models to new protein. Further, it can be combined with other methods for better performance. The limitation of the method is the usage of only 30 specific folds for discrimination.

## Conclusion

In this paper, we have proposed a simple method for discriminating 30 folding types of globular proteins. Interestingly, the simplest method is the best method for the truly complicated problems. Although complicated methods have several possibilities for tuning they generate over fitting to the data set. Further, the method proposed in this work is better than or comparable to other complicated methods, such as, neural networks and support vector machines proposed in the literature for discriminating folding types. In addition, our method has several advantages including the less computational time and classifying the folds at a single run rather than pair-wise comparisons. We have developed a web server [24], which takes the amino acid sequence as the input and displays the folding type in the output. The main limitation of the method is that its application is restricted to 30 folds considered in this work. However, the approach can be extended to other folds when significant representatives are available.

## Methods

### Dataset

We have used a dataset of 1612 globular proteins belonging to 30 major folding types obtained from SCOP database [25] for recognizing protein folds. This dataset has been constructed with the following criteria: (i) there should be at least 25 proteins in each fold and (ii) the sequence identity between any two proteins is not more than 25%. The amino acid sequences of all the proteins are available at [24].

### Linear discriminant analysis

We have employed LDA in this work and a brief description is given below. First, we compute the amino acid occurrence of each protein,

**n**_*i *_≡ (*n*_*i*1_, *n*_*i*2_, ..., *n*_*ij*_, ..., *n*_*i*20_),

where i is the number of protein; *n*_*i*1_, *n*_*i*2 _etc. represents the number of amino acids of each type (Ala, Arg etc.) in *i*th protein. Then LDA tries to maximize

$$\eta^{2}=\frac{S_{B}}{S_{T}}.$$

*S*_*B*_(*S*_*T*_) is the summation of squared distance between the center of mass of all proteins and that within fold (coordinate of each protein) along axis *z*, i.e.,

$$S_{B}\equiv \sum_{k=1}^{K}N_{k}{\left(\bar{z}-z_{k}\right)}^{2}$$

$$S_{T}\equiv \sum_{i}{\left(\bar{z}-z_{i}\right)}^{2},$$

where *K *is the number of folds, *N*_*k *_is the number of proteins belonging to *k*th fold and $\bar{z}$ is the center of mass along the axis *z*, and *z*_*k *_is that within *k*th fold, i.e.,

$$\bar{z}\equiv \frac{1}{N}\sum_{i}z_{i}$$

$$z_{k}\equiv \frac{1}{N_{k}}\sum_{i^{\prime }=1}^{N_{k}}z_{i^{\prime }},$$

where *i' *is the *i' *th protein within the *k*th fold. *z*_*i *_is the linear combination of *n*_*ij *_with the set of coefficients **a **≡ (*a*_0_, *a*_1_, ..., *a*_*j*_, ... *a*_20_),

$$z_{i}\equiv a_{0}+\sum_{j}a_{j}n_{ij}$$

Hence, LDA tries to find **a **which maximizes *η*^2^. In total, we can get 20 kinds of *z*_*i*_s which are orthogonal to each other, and discrimination is done based on these *z*_*i*_s. In addition one can introduce weights *W*_*k *_for each group using the equation:

(1) $$S_{B}\equiv \sum_{k=1}^{K}W_{k}{\left(\bar{z}-z_{k}\right)}^{2}$$

The discrimination is done with Bayesian scheme employing Gaussian kernel. Proteins in each fold are assumed to distribute in amino acid occurrence space obeying Gaussian distribution whose center is the mean occurrence within each fold and variance is computed along the 20 kinds of *z *coordinates. Then the fold with maximum probability is used assign the folding type of a protein. The probability of each fold may also be used to find other probable folds for a specific sequence.

We have used lda module in MASS library of R [26] and the computational time is less than few seconds using Intel Pentium M processor (1.10 GHz) and 1 GB memory.

### Scoring

In this paper, we employed four performances to validate the results. We computed TP_*k *_which is the number of proteins being correctly discriminated to be in *k*th category (e.g., fold). We have also computed FP_*k *_(FN_*k*_), which is the number of proteins which are incorrectly discriminated as being (not being) in *k*th category. Then we defined sensitivity (or recall), Precision, and F1 as

$$\text{Sensitivity (Recall)}=\frac{{\text{TP}}_{k}}{{\text{TP}}_{k}+{\text{FN}}_{k}}$$

$$\text{Precision}=\frac{{\text{TP}}_{k}}{{\text{TP}}_{k}+{\text{FP}}_{k}}$$

$$\text{F}1=\frac{2\times \text{Precision}\times \text{Sensitivity}}{\text{Precision}\times \text{Sensitivity}}.$$

For validating whole data set we have taken the category average,

$$\frac{1}{K}\sum_{k=1}^{K}\left(\text{Performance}\right),$$

where (Performance) is sensitivity/precision/F1. For some cases denominator of precision and/or F1 will be zero and we excluded these categories to compute the average.

The accuracy is defined as,

$$\text{Accuracy}=\frac{\sum_{k=1}^{K}{\text{TP}}_{k}}{N}.$$

N is the total number of proteins.

## Availability and requirements

Project name: PROLDA

Project home page: http://www.granular.com/PROLDA/

Operating systems : Platform independent

Programing languages : R [26]

Licence: GNU GPL

Any restrictions to use non-academics: none

## Authors' contributions

YhT coded the program, carried out most of the calculations and constructed the prediction server. MMG directly supervised the work and provided the dataset of amino acid sequences. All authors contributed in the preparation of the manuscript, read and approved it.
